# What is the value of musculoskeletal ultrasound in patients presenting with arthralgia to predict inflammatory arthritis development? A systematic literature review

**DOI:** 10.1186/s13075-018-1715-8

**Published:** 2018-10-11

**Authors:** Rosaline van den Berg, Sarah Ohrndorf, Marion C. Kortekaas, Annette H. M. van der Helm-van Mil

**Affiliations:** 1 Department of Rheumatology, Erasmus Medical Center, Rotterdam, The Netherlands; 2 Department of Rheumatology, Leiden University Medical Center, Leiden, The Netherlands; 3 Department of Rheumatology and Clinical Immunology, Charité – Universitätsmedizin Berlin, Berlin, Germany

**Keywords:** Arthralgia, Ultrasound, Rheumatoid arthritis

## Abstract

**Objective:**

Musculoskeletal ultrasound (US) is frequently used in several rheumatology practices to detect subclinical inflammation in patients with joint symptoms suspected for progression to inflammatory arthritis. Evaluating the scientific basis for this specific US use, we performed this systematic literature review determining if US features of inflammation are predictive for arthritis development and which US features are of additive value to other, regularly used biomarkers.

**Methods:**

Medical literature databases were systematically searched up to May 2017 for longitudinal studies reporting on the association between greyscale (GSUS) and Power Doppler (PDUS) abnormalities and inflammatory arthritis development in arthralgia patients. Quality of studies was assessed by two independent reviewers using a set of 18 criteria. Studies were marked high quality if scored ≥ 80.6% (which is the median score). Best-evidence synthesis was performed to determine the level of evidence (LoE). Positive and negative likelihood ratios (LR+, LR−) were determined.

**Results:**

Of 3061 unique references, six fulfilled inclusion criteria (three rated high quality), of which two reported on the same cohort. Heterogeneity in arthralgia populations, various US machines and scoring systems hampered the comparability of results. LoE for GSUS as predictor was limited and moderate for PDUS; LoE for the additive value of GSUS and PDUS with other biomarkers was limited to moderate. Estimated LR+ values were mostly < 4 and LR− values > 0.5.

**Conclusions:**

Data on the value of GSUS and PDUS abnormalities for predicting inflammatory arthritis development are sparse. Although a potential benefit is not excluded, current LoE is limited to moderate. Future studies are required, preferably performed in clearly defined, well-described arthralgia populations, using standardized US acquisition protocols and scoring systems.

**Electronic supplementary material:**

The online version of this article (10.1186/s13075-018-1715-8) contains supplementary material, which is available to authorized users.

## Background

The development of rheumatoid arthritis (RA) is supposed to consist of several stages: a) genetic risk factors for RA; b) environmental risk factors for RA; c) systemic autoimmunity associated with RA; d) symptoms without clinical arthritis; e) unclassified arthritis (UA); f) RA [1]. The phase of arthralgia preceding clinical arthritis (phase d) is of particular interest since it is hypothesized that disease-modifying treatment initiated in this phase might result in better disease outcomes than when initiated in the phases of UA and RA [2]. However, musculoskeletal symptoms such as arthralgia are prevalent, and arthralgia is frequently not related to imminent RA. In order to identify arthralgia patients at risk for RA, different strategies can be undertaken, such as selecting arthralgia patients based on clinical features associated with RA development, using autoantibody tests or imaging to detect subclinical inflammation, or a combination of these.

Musculoskeletal ultrasound (US) is a frequently used imaging modality as it is fast, easy to apply, and readily accessible. Although US is frequently used in patients presenting with arthralgia (as also proposed in an algorithm for the pragmatic use of US [3]) in several rheumatology practices, we questioned what the scientific basis is to use US as a predictor for future inflammatory arthritis development. Therefore, we systematically studied the literature to determine if US features of inflammation are predictive for inflammatory arthritis development and, if so, to determine which US features are of additive value to other regularly used biomarkers, with the ultimate goal of obtaining evidence-based information on the value of US in patients presenting with arthralgia.

## Methods

### Systematic literature search

The PRISMA guidelines were followed [4]. Search strategies were built in collaboration with an experienced librarian (WB) and executed in electronic medical literature databases (Embase.com, Medline Ovid, Web of Science, Scopus, Cochrane Central, Google Scholar) up to 11 May 2017 (complete searches in Additional file 1: File S1). Reference lists of the included papers were checked for additional papers and unpublished and ongoing trials were identified using the World Health Organization (WHO) International Clinical Trials Registry Platform (ICTRP) search portal (http://apps.who.int/trialsearch/) and ClinicalTrials.gov (http://clinicaltrials.gov).

### Selection of studies based on inclusion and exclusion criteria

Two reviewers (SO, RvdB) assessed each title for suitability for inclusion in this review, according to predetermined inclusion and exclusion criteria. Next, abstracts were retrieved for detailed review and, finally, full-text papers were assessed if further information was required. Papers not addressing the topic of interest were excluded and reasons for exclusion recorded.

From the total number of studies identified by the database search, studies were included if the following inclusion criteria were met: 1) investigation of subjects without clinical arthritis, suffering from arthralgia, regardless of rheumatoid factor (RF) and anti-citrullinated protein antibody (ACPA) status or ACPA+ musculoskeletal symptoms; 2) investigation of small hand and/or feet joints of subjects using US; 3) joints and/or tendons were assessed for inflammatory features (GS synovial hypertrophy and/or PDUS); 4) subjects were followed prospectively; 5) development of (persistent) inflammatory arthritis or RA was defined as outcome. Studies about other inflammatory joint conditions, animal studies, reviews, letters to the editor, case reports, case series, commentaries, guidelines, editorials, abstracts, study populations < 18 years of age, and studies in languages other than English, Dutch, and German were excluded.

### Data extraction

The two reviewers independently assessed the full texts of the included studies using a predefined sheet to extract data about: 1) study population (number of patients, age, gender, symptom duration); 2) follow-up period; 3) musculoskeletal US equipment (producer, transducer, machine setting, mode (GSUS/PDUS); 4) US acquisition (number and type of examined joints, examined pathology, scoring method, potential used cut-off); 5) longitudinal outcome.

Data from univariable analyses were extracted to answer the first aim; data from multivariable analyses were extracted to answer the second aim on added value.

### Quality assessment and analyses

Due to heterogeneity of the studies, it was not possible to perform meta-analyses and calculate pooled effect estimates. Therefore, we performed a best-evidence synthesis based on the guidelines on systemic review of the Cochrane Collaboration Back and Neck (CBN) Group [5], a method summarizing the level of evidence (LoE) in observational studies if study population, outcomes and data analyses are heterogenic (Additional file 1: Table S1). LoE is based on presence of statistical significance, which depends on sample sizes, taking into account the quality of the studies. Quality of the studies was evaluated by the two reviewers individually, using a set of 18 criteria based on previous systematic reviews in prognostic factors in the field of musculoskeletal disorders [2, 6]. This list included seven criteria specifically for the use of US, of which three were considered mandatory (Additional file 1: Table S2). A study was considered high quality if all three mandatory criteria were fulfilled and the total score was ≥ 80.6% (median of quality scores obtained in this review).

Positive and negative likelihood ratios (LR+ and LR−, respectively) and positive and negative predictive values (PPV and NPV, respectively) were calculated based on presented data regarding outcome (using the presented follow-up duration (Table 1)) to evaluate the predictive accuracy. Also, due to heterogeneity, no summary estimates were calculated.

**Table 1:** Overview of selected studies

Study	Study population	N	Female (%)	Age (years; mean (±SD) *or* median (IQR))	Symptom duration at inclusion (mean (±SD) or median (IQR))	Outcome of relevance	Mean follow-up duration (months; mean (±SD) or median (IQR))	N (%) patients with outcome	Duration until diagnosis/ outcome (months)	Univariable	Adjustment factors	Multivariable
**Rakieh et al. 2015** [11]	**ACPA+ patients with MSK symptoms** **(primary and secondary care)**	**100**	**69**	**51.2 ± 11.9**	**22.7 (8.2–42.4) months**	**IA**	**19.8 (7.6–34.4)**	**50 (50.0)**	**7.9 (3.2–14.5)**	**PDUS≥ 1: HR 1.88 (1.07–3.29)**	**Tenderness small joints** **Morning stiffness ≥ 30 min** **High ++ RF and/or ACPA**	**PDUS ≥ 1: HR 1.51 (0.83–2.74)** ^**¥**^
**Nam et al. 2016** [10]	**ACPA+ patients with MSK symptoms** **(primary and secondary care)**	**136**	**73.7**	**51.3 ± 12.4**	**17.2 (7.0–33.4) months**	**IA**	**28.1 (range 4.7–79.6) for non-progressors**	**57 (41.9)**	**18.3 (range 0.1–79.6)**	**GSUS ≥ 2: HR 2.8 (0.4–20.3)** **PDUS ≥ 1: HR 1.6 (0.9–3.2)**	**None**	**ND**
**van der Ven et al. 2017** [8]	Inflammatory arthralgia in > = 2 painful joints (hands, feet, shoulders), plus 2 additional criteria* **(secondary care)**	**174**	**83**	**45.0 ± 11.3**	**7.0 ± 3.1 months**	**IA**	**12**	**31 (17.8)**	**Within 1 year; not specified**	**GSUS ≥ 2 and/or PDUS ≥ 1** ^**⌃**^ **:** **OR 3.03 (1.69–5.41)** **PDUS ≥ 1:** **OR 3.12 (1.61–6.03)**	**GSUS ≥ 2 and/or PDUS ≥ 1** ^**⌃**^ **:** **Age** **Morning stiffness > 30 min** **ACPA** **PDUS ≥ 1:** **Age** **Morning stiffness > 30 min**	**GSUS ≥ 2 and/or PDUS ≥ 1** ^**⌃**^ **:** **OR 2.65 (1.44–4.88)** **PDUS ≥ 1:** **OR 3.44 (1.71–6.95)**
van de Stadt et al. 2010 [12]	Arthralgia with RF+ and/or ACPA+ (secondary care)	192	72	47 ± 11	12 (9–36) months	Arthritis	26 (range 6–54)	45 (23.4)	11 ± 9	Synovitis: OR 1.41 (0.54–3.65) PDUS: OR 1.54 (0.67–3.54) Effusion: OR 2.05 (0.80–5.27) Tenosynovitis: OR 1.50 (0.44–5.11)	None	ND
Pratt et al. 2013 [9]	Inflammatory arthralgia (secondary care)	379	72	51 (36–66)	20 (10–34) weeks	Persistent IA^ǂ^	27 (range 12–44)	162 (42.7)	NP	NP	Age Symptom duration Swollen joint count CRP ACPA ESR	Grade 1 GSUS synovitis in ≥ 3/16 joints: OR 4.91 (2.32–10.4)
Zufferey et al. 2017 [7]	ACPA- and RF- inflammatory polyarthralgia > 6 weeks (secondary care)	80	77	51 ± 14	NP	RA	18 ± 7	7 (8.8)	18	NP	Gender Elevated CRP	SONAR > 8/22^⌃^: OR 7.45 (1.19–42.8) US score ≥ 2 joints with grade ≥ 2 synovitis^⌃^: OR 10.1 (1.1–49)

Studies marked in bold are scored as high-quality (high-quality study > 80% (which is the median of all quality scores))

*GSUS* greyscale ultrasound, *NA* not applicable, *ND* not done, *NP* not presented, *NPV* negative predictive value, *PPV* positive predictive value, *PDUS* power Doppler ultrasound, *IA* inflammatory arthritis, *MSK* musculoskeletal

^*^Morning stiffness for more than 1 h, unable to clench a fist in the morning, pain when shaking someone’s hand, pins and needles in the fingers, difficulty wearing rings or shoes, family history of RA and/or unexplained fatigue for < 1 year

^ǂ^Persistent IA was defined as RA, psoriatic arthritis, enteropathic arthritis, ankylosing spondylitis, undifferentiated spondyloarthritis, connective tissue disease, “self-limiting inflammatory/reactive arthritis” warranting DMARD treatment and other inflammatory arthritides

^¥^In the PDUS model corrected for tenderness small joints, morning stiffness ≥ 30 min, high ++ RF and/or ACPA

^§^ One or more swollen joint on physical examination

^⌃^See Table 2 for a detailed description of the cut-offs and thresholds used to define a positive US

## Results

### Selection and inclusion of articles

In total, 5028 titles were identified and, after removing duplicates, 3061 unique references were screened (Additional file 1: Figure S1). After detailed review, six full-text papers fulfilled the inclusion and exclusion criteria (Table 1) [7–12], of which two studies reported on the same cohort [10, 11]. One of them reports on dichotomous PDUS results only and the other presents PDUS and GS synovial hypertrophy results for various cut-offs.

### Quality assessment

The two reviewers rated 108 items and agreed on 98 (91.6%); disagreement on items was solved by discussion (Additional file 1: Table S3). All six included studies fulfilled the three mandatory criteria. Median quality score was 80.6% (range 61.1–83.3%). Two of the three high-quality papers described the same cohort [8, 10, 11].

### Study characteristics

The number of included patients varied between 80 and 379; the majority were female (69–83%) aged > 50 years. None of the studies had stringent inclusion criteria with respect to symptom constitution. The cohort described in the papers by Nam et al. [10] and Rakieh et al. [11] included ACPA+ patients with new onset musculoskeletal symptoms from primary care physician clinics and the rheumatology early arthritis clinic in Leeds. In the study of Van der Ven et al. [8], patients with inflammatory joint complaints involving at least two joints in the hands, feet, or shoulders for < 1 year which could not be explained by other conditions were included if they had also at least two of the following criteria: morning stiffness for > 1 h, unable to clench a fist in the morning, pain when shaking someone’s hand, pins and needles in the fingers, difficulties wearing rings or shoes, family history of RA, and/or unexplained fatigue. In the paper by Zufferey et al. [7], ACPA- and RF-negative patients with polyarthralgia for > 6 weeks with an inflammatory or mixed (mechanical and inflammatory) character referred by their general practitioner or rheumatologist were included. Van de Stadt et al. [12] recruited ACPA+ and/or RF+ patients with arthralgia, defined as “non-traumatic pain in any joint”, at rheumatology clinics in Amsterdam after referral by their general practitioner. Patients presenting with new-onset arthralgia to the Newcastle Early Arthritis Clinic were included in the study by Pratt et al. [9], but no description of arthralgia was provided.

Symptom duration at inclusion varied between 6 weeks and 23 months (Table 1). Patients were followed for > 12 months in all studies (range 12–28 months). Three studies included only ACPA+ and/or RF+ patients [10–12]; one study only ACPA- and RF-negative patients [7] and the remaining studies included both ACPA+ and/or RF+ and arthralgia negative patients [8, 9].

### Acquisition of ultrasound

US specifications are presented in Table 2. Three studies used a transducer with 12 or 13 MHz as maximum [7, 9, 12]. Various US machines were used, various scoring systems with various definitions of pathology were used to grade synovitis [13–20], and the number of examined joints varied (range 16–32). In one study only tender joints were scanned [12]. Four studies reported on both GS synovial hypertrophy and PDUS [8–10, 12], one only on GS synovial hypertrophy [7], and one only on PDUS [11]. Only one study scored the presence of tenosynovitis (GSUS) [12]. All studies except one [10] used a cut-off to define a positive “inflammation US score”, yet the definitions varied (Table 2).

**Table 2:** Specification of US in selected study

Study	Machine	Probe	Mode	Synovitis (scoring method)	Tenosynovitis (scoring method)	Erosion	Locations scanned	One side (1)/both sides (2)	Total number of joints	Volar/dorsal side	Cut-off/threshold def. “inflammation US score”	Positive “inflammation US score”, % total group (progressors, non-progressors)
**Rakieh et al. 2015** [11]	**Philips ATL HDI 5000**	**12–5 MHz and 8–15 MHz**	**PDUS**	**Yes (0–3)** [16, 19]	**ND**	**ND**	**Wrist** **MCP I-V** **PIP I-V**	**2**	**22**	**NP**	**PDUS ≥ 1**	**33.0 (44.0, 22.0)**
**Nam et al. 2016** [10]	**Philips ATL HDI 5000 and General Electric S7**	**5–12 and 8–15 MHz (Philips); 6–15 MHz (GE)**	**GSUS and PDUS**	**Yes (0–3; for both GSUS and PDUS)** [22]	**ND**	**Yes (0/1)**	**Wrist** **MCP I-V** **PIP I-V** **MTP I-V**	**2**	**32**	**Dorsal**	**None**	**GSUS = 0: 4.4 (1.8, 6.3)** **GSUS = 1: 27.9 (21.1, 32.9)** **GSUS ≥ 2: 67.6 (77.2, 60.8)** **PDUS = 0: 66.9 (50.9, 78.5)** **PDUS = 1: 18.4 (22.8, 15.2)** **PDUS = 2: 14.7 (26.3, 6.3)** **ERO = 0: 79.4 (64.9, 89.9)** **ERO = 1: 20.6 (35.1, 10.1)**
**van der Ven et al. 2017** [8]	**Mylab 60 (Esaote, Genoa, Italy)**	**10–18 MHz**	**GSUS and PDUS**	**Yes (0–3; for both GSUS and PDUS)** [15]	**ND**	**ND**	**Wrist** **MCP II-V** **PIP II-V** **MTP II-V**	**2**	**26**	**Dorsal**	**a. Positive synovitis: GSUS ≥ 2 and/or PDUS ≥ 1** **b. PDUS score: ≥ 1**	**a. 35.6 (54.8, 31.5)** **b. 14.9 (29.0, 11.9)**
van de Stadt et al. 2010 [12]	Acuson Antares, premium edition (Siemens, Malvern, PA, USA)	5–13 MHz	GSUS and PDUS	Yes (0–3; for both GSUS and PDUS) [13]	Yes (0–3)	ND	Only tender joints*	2	NA	Volar	PDUS ≥ 1 Joint effusion, synovitis, tenosynovitis ≥ 2	GSUS synovitis ≥ 2: 12.5 (15.6, 11.6) GSUS effusion ≥ 2: 11.5 (17.7, 9.5) PDUS ≥ 1: 17.2 (22.2, 15.6) Tenosynovitis ≥ 2: 6.8 (8.9, 6.1)
Pratt et al. 2013 [9]	Aplio Diagnostic Ultrasound System (Toshiba Medical Systems Corporation, Tochigi-Ken, Japan)	12 MHz	GSUS and PDUS	Yes (0–3; for both GSUS and PDUS) [13–15, 20]	ND	Yes (0–3)	MCP II-IV PIP II-IV MTP I-II	2	16	Dorsal and volar	GSUS: a. sum score ≥ 2; b. sum score/6 joints (worst hand) ≥ 2; c. number of joints ≥ 1: ≥ 3. PDUS: d. sum score ≥ 1; e. number of joints ≥ 1: ≥ 2	a. 35.1 (56.2, 19.4) b. 29.6 (48.8, 15.0) c. 30.1 (50.6, 14.7) d. 29.0 (46.9, 15.7) e. 16.9 (29.6, 7.4)
Zufferey et al. 2017 [7]	Philips HD 11	7–13 MHz	GSUS	Yes (0–3) [17, 18]	ND	ND	Wrist MCP II-V PIP II-V Elbows Knees	2	22	NP	a. B-mode score > 8 (of total possible score of 66). b. ≥ 2 joints (of total number of 22 joints) with grade ≥ 2 synovitis [18]	a. 21.3 (57.1, 17.8) b. 25.0 (71.4, 20.5)

Studies marked in bold are scored as high-quality (high-quality study > 80% (which is the median of all quality scores))

*ERO* erosions, *GSUS* greyscale ultrasound, *MCP* metacarpophalangeal joint, *MHz* megahertz, *MTP* metatarsophalangeal joint, *NA* not applicable, *ND* not done, *NP* not presented, *PIP* proximal interphalangeal joint, *PDUS* power Doppler, *US* ultrasound

*Tender joints at physical examination were scanned, otherwise joints that were painful by history were scanned. For MCP, PIP, and MTP joints the directly adjacent joints in the same joint group as the painful joints were scanned

Two studies reported on inter-observer reliability, which was moderate (kappa = 0.56 for GS synovial hypertrophy) to substantial (kappa = 0.64 for PDUS) [9] in one study, and fair (kappa = 0.22 for effusion) to moderate (kappa = 0.47 for synovitis) and substantial (kappa = 0.67 for PDUS) in another study [12], yet good in terms of overall percentage agreement (88–92%).

### Outcome

Outcome was defined as RA (ACR/EULAR 2010 criteria [21]) in one study and (persistent) (inflammatory) arthritis in the remaining five. Outcome was reached in 8.8–50.0% of patients; frequency was lowest in ACPA-/RF-negative populations and highest in ACPA+/RF+ populations. Duration until outcome was reached varied between 7.9 and 18.3 months and was not specified in two studies (Table 1).

### LoE of GSUS and PDUS abnormalities as predictor for arthritis development

The prevalence of different US features varied per patient group and cut-off used. For GS synovial hypertrophy it ranged from 11.6 (GSUS ≥ 2 in patients without arthritis development) to 77.2% (GSUS ≥ 2 in patients that developed arthritis); for PDUS from 6.3 (PDUS = 2 in patients without arthritis development) to 44.0% (PDUS ≥ 1 in patients that developed arthritis) (Table 2). The prevalence of tenosynovitis ranged from 6.1 (GSUS ≥ 2 in patients without arthritis development) to 8.9% (GSUS ≥ 2 in patients with arthritis development).

#### GS synovial hypertrophy

One high-quality and one low-quality study reported a non-statistically significant association between GS synovial hypertrophy and arthritis development (HR 2.8 [95% CI 0.4–20.3] and (OR 1.41 [95% CI 0.54–3.65], respectively) [10, 12]. One other high-quality study reported a statistically significant association (OR 3.03 [95% CI: 1.69–5.41]) for a “positive US” defined as GSUS ≥ 2 and/or PDUS ≥ 1 [8]. Hence, LoE with regard to the predictive value of GSUS is limited.

#### PDUS synovitis

Two high-quality studies reported a statistically significant association between PDUS and arthritis development (OR 3.12 [95% CI 1.61–6.03] [8], HR 1.88 [95% CI 1.07–3.29] [11]). The third high-quality study (performed in the same cohort as [11]) reported a non-statistically significant association (HR 1.6 [95% CI 0.9–3.2]) [10]; thus the statistically significant association found in the first 100 patients was lost after inclusion of additional patients. A low-quality study reported a non-significant association as well (OR 1.54 [95% CI 0.67–3.54]) [12]. Hence, LoE with regard to the predictive value of PDUS is moderate.

#### Tenosynovitis

One low-quality study evaluated tenosynovitis and found no statistically significant association with arthritis development (OR 1.50 [95% CI 0.44–5.11]) [12]. Hence, LoE with regard to the predictive value of tenosynovitis is insufficient.

### LoE of GSUS and PDUS abnormalities being additive to other biomarkers

Three studies investigated the association of GS synovial hypertrophy with arthritis development, correcting for different biomarkers (Table 1). Two low-quality studies reported statistically significant associations of GS synovial hypertrophy and arthritis development (OR 4.91 [95% CI 2.32–10.4]), OR 7.45 [95% CI 1.19–42.8], and OR 10.1 [95% CI 1.1–49] [7, 9]. One high-quality study reported a statistically significant association of a “positive US” (GSUS ≥ 2 and/or PDUS ≥ 1; OR 2.65 [95% CI 1.44–4.88]) [8]. Hence, LoE with regard to the question of whether GS synovial hypertrophy may have value in predicting arthritis development, additive to regularly assessed biomarkers, is moderate.

Likewise, two studies performed multivariable analysis with PDUS. After correction for (different) biomarkers (Table 1), one high-quality study reported a statistically significant association (OR 3.44 [95% CI 1.71–6.95]) [8]. The other high-quality study reported a non-significant association (HR 1.51 [95% CI 0.83–2.74]) [11]. Hence, LoE of the value of PDUS in addition to other biomarkers is limited.

The value of tenosynovitis (GS/PD) in addition to other biomarkers was not investigated.

### Positive and negative likelihood ratios and absolute risks

**Fig. 1:**
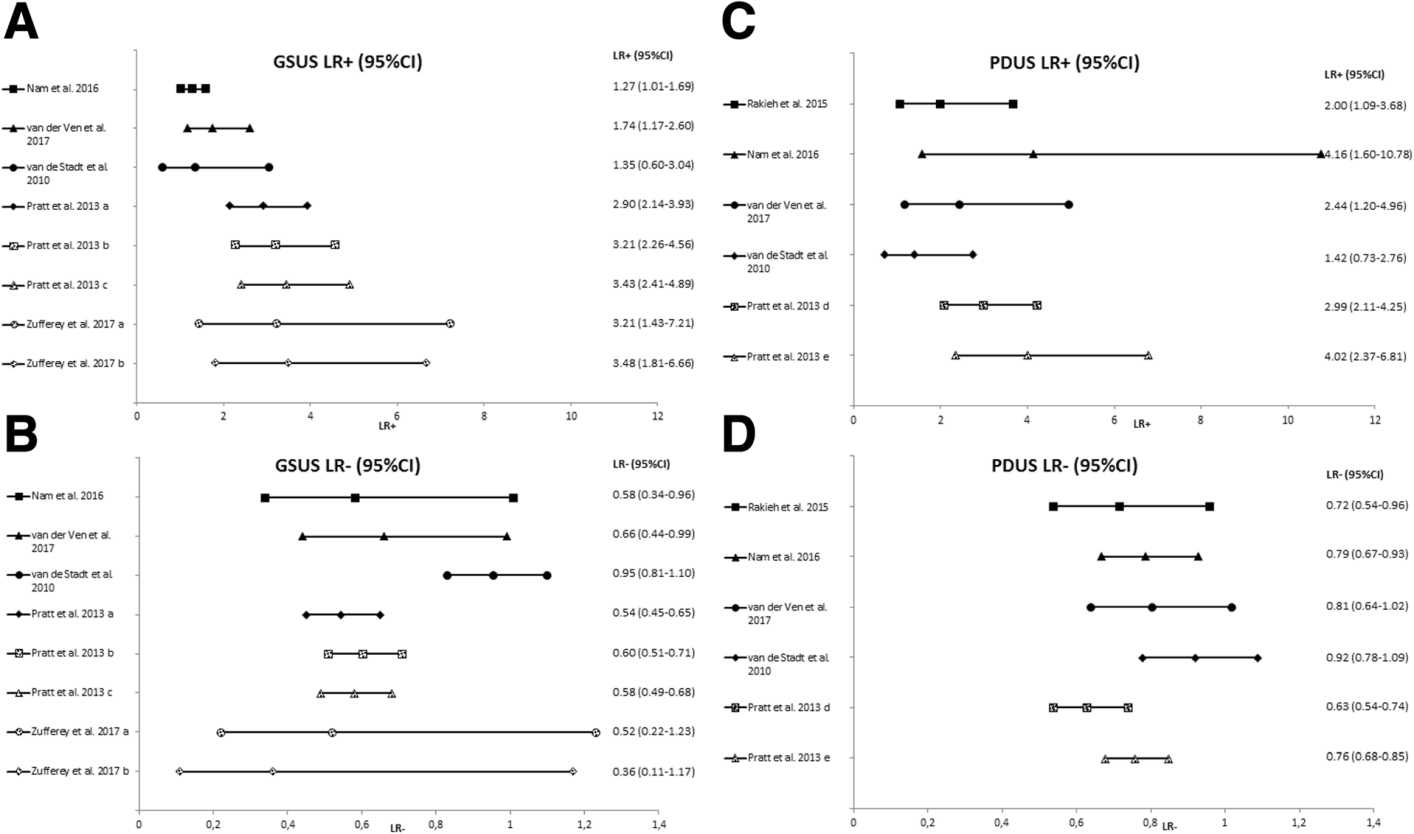
Forest plots of LR+ and LR− for GSUS (**a**, **b**) and PDUS (**c**, **d**). LR+ = positive likelihood ratio; LR− = negative likelihood ratio. *GSUS* greyscale ultrasound, *PDUS* power Doppler ultrasound. Some studies used different cut-offs and are presented two or three times in this figure. Pratt: a GSUS sum score ≥ 2; b GSUS sum score/6 joints (worst hand) ≥ 2; c GSUS number of joints ≥ 1: ≥ 3; d PDUS sum score ≥ 1; e PDUS number of joints ≥1: ≥ 2. Zufferey: a B-mode score > 8 (of total possible score of 66); b ≥ 2 joints (of total number of 22 joints) with grade ≥ 2 synovitis [18]. Likelihood ratio values between 0 and 1 decrease the probability of disease; values greater than 1 increase the probability of disease. An LR of 1 does not influence the probability. In general, an LR+ of 2 results in an approximate change of + 15% in post-probability; an LR+ of 5 in an approximate change of + 30% and an LR+ of 10 in an approximate change of + 45%. An LR− of 0.5 results in an approximate change of − 15% in post-probability; an LR− of 0.2 in an approximate change of − 30% and an LR− of 10 in an approximate change of − 45%. These estimations are accurate for pre-test probabilities between 10% and 90% [23]

Calculated LRs varied and confidence intervals (CIs) were wide. For GS synovial hypertrophy, LR+ ranged from 1.27–3.48 and LR− ranged from 0.36–0.95. For PDUS, LR+ ranged from 1.42–4.16 and LR− ranged from 0.63–0.92 (Fig. 1 and Additional file 1: Table S4).

Predictive values are directly proportional to disease prevalence. Percentages of patients that developed arthritis varied between 8.8 and 50%; thus, prior risks for not progressing were 50–91.2%. We calculated the increase in the absolute risks of inflammatory arthritis provided by US-detected abnormalities by comparing PPV and NPV with prior risks (Additional file 1: Table S4). Overall, PPVs were low or moderate (23.5–71.9% for GS synovial hypertrophy; 30.3–75% for PDUS) and the increase in absolute risks in US-positive patients ranged from 5.8–29.2% (GS synovial hypertrophy) and 6.9–33.1% (PDUS). NPVs were higher (68.9–96.7% for GS synovial hypertrophy; 58.2–85.1% for PDUS), but the gain in relation to prior risk of not progressing to arthritis was relatively small (0.8–12.5% for GS synovial hypertrophy; 2.9–13.9% for PDUS). Thus, NPVs were largely explained by prior risks of not developing inflammatory arthritis.

## Discussion

The aim of this systematic literature review was to determine if US features of inflammation are predictive for inflammatory arthritis development and, if so, which US features are of additive value to other regularly used biomarkers. LoE for GS synovial hypertrophy as predictor for arthritis was limited and moderate for PDUS. LoE for the additive value of GS synovial hypertrophy and PDUS with other regularly used biomarkers was limited to moderate. Additionally, there was insufficient data on the value of US-detected tenosynovitis. Thus, there is a discrepancy between the frequent use of US in arthralgia patients to search for subclinical inflammation (which, if present, is generally considered a sign of imminent RA) in several rheumatology practices and the absence of strong scientific evidence on its prognostic value.

The limited/moderate LoE might be explained by relatively low number of studies and the presence of different types of heterogeneity. Only six studies were included in this systematic literature review, of which two described the same cohort. The number of included patients per study was rather low, influencing the power to achieve statistical significance. Furthermore, heterogeneous arthralgia populations (seropositive arthralgia, seronegative arthralgia, ACPA+ patients with unspecific musculoskeletal (MSK) symptoms) were studied in different settings (primary and/or secondary care), with slightly differently defined outcomes ((persistent) (inflammatory) arthritis, RA), contributing to the various ranges of frequencies of outcome (8.8–50%).

Moreover, the US acquisition protocol, definitions of pathology, and scoring systems varied, although all followed internationally recognized recommendations and scoring systems [13–20]. Only very recently, EULAR/OMERACT published a standardized, consensus-based semi-quantitative scoring system for GS synovial hypertrophy and PDUS (separately and combined) [24, 25], but this was not available when the studies included in this review were executed.

Other sources of heterogeneity were the selection of assessed joints, whether they were scanned from a volar or dorsal aspect, and the fact that different machines were used. It is known that the diverse machines have a wide variation in sensitivity to pick up inflammation, especially with regard to Doppler modalities [26]. Three studies used a transducer with 12 or 13 MHz as maximum, while higher frequencies are recommended especially for scanning small hand joints. Ideally, in order to arrive at a higher LoE, future studies should be performed in more homogeneous arthralgia populations (e.g., fulfilling the EULAR definition of arthralgia at risk for RA [27]), using the same scan and scorings protocols (e.g., EULAR/OMERACT [24, 25]).

Another issue is the definition of a “positive US”. Different cut-offs were applied and none of the studies included information on US findings in healthy volunteers. It has been shown that a cut-off incorporating such findings increased the prognostic value for the use of MRI in arthralgia patients [28]. Also US “inflammatory features” can be detected in healthy volunteers, especially in certain joints and increasing with age [29–36]. Whether incorporating age-dependent US reference values might increase the predictive value of US remains to be determined.

There was insufficient data to determine whether US-detected tenosynovitis is an (important) predictor of arthritis development, which is the case for MRI-detected subclinical tenosynovitis (which is an even stronger predictor than MRI-detected subclinical synovitis or bone marrow edema) [37]. Therefore, the potential of US-detected tenosynovitis requires further investigation.

We sought to explore the value of US abnormalities in addition to other frequently used predictors of arthritis development. Some studies performed multivariable analyses but adjusted for different variables; hence, the results of these multivariable analyses could not be directly compared. Further studies on this subject are needed, also using methods such as net reclassification index.

Best-level evidence synthesis focuses on statistical significance. Since this is not directly applicable for clinical practice, we also expressed prognostic accuracy using LRs. Estimated LR+ values were mostly < 4 and LR− values > 0.5, some with wide CIs, indicating that the post-test probability was altered to only a small degree. This was also observed when we calculated increases in absolute risks (comparing pre-test with observed post-test risks). Although absolute NPVs were higher than PPVs, and seemingly more informative, this was caused by the prior risks, which were relatively low. Our comparison of pre-test and post-test risks suggested that US is slightly more helpful in “ruling in” than “ruling out” imminent inflammatory arthritis.

## Conclusions

US is frequently used in arthralgia patients in several rheumatologic practices, and although some studies have suggested a potential benefit of US, the current LoE is limited to moderate at best, due to heterogeneity of studies and lack of replication. Yet, there is a strong need for validation of results in future US studies, preferably performed in clearly defined, well-described arthralgia patients. The EULAR definition of arthralgia suspicious for progression to RA might be used to this end.

## Additional file

Additional file 1: Overview of literature research, Best-evidence synthesis, Criteria and scores of the quality assessment, Calculated PPVs & NPVs and increase in absolute risks, Search strategies for each database. (DOCX 85 kb)

## Abbreviations

ACPA: Anti-citrullinated protein antibody
ACR: American College of Rheumatology
CI: Confidence interval
EULAR: European League Against Rheumatology
GS: Greyscale
GSUS: Greyscale ultrasound
HR: Hazard ratio
ICTRP: International Clinical Trials Registry Platform
LoE: Level of evidence
LR−: Negative likelihood ratio
LR+: Positive likelihood ratio
MHz: Megahertz
MRI: Magnetic resonance imaging
NPV: Negative predictive value
OMERACT: Outcome measures in rheumatology
OR: Odds ratio
PD: Power Doppler
PDUS: Power Doppler ultrasound
PPV: Positive predictive value
RA: Rheumatoid arthritis
RF: Rheumatoid factor
UA: Unclassified arthritis
US: Ultrasound
WHO: World Health Organization
