# Supplementary material for: What is the value of musculoskeletal ultrasound in patients presenting with arthralgia to predict inflammatory arthritis development? A systematic literature review
Source: Arthritis Res Ther. 2018 Oct 11;20:228. doi: 10.1186/s13075-018-1715-8 (PMC6235211; doi:10.1186/s13075-018-1715-8)
Supplement: Supplementary file 1 — Overview of literature research, Best-evidence synthesis, Criteria and scores of the quality assessment, Calculated PPVs & NPVs and increase in absolute risks, Search strategies for each database. (DOCX 85 kb) [file 13075_2018_1715_MOESM1_ESM.docx]

**Additional file 1**

**Figure S1: Overview of literature research**
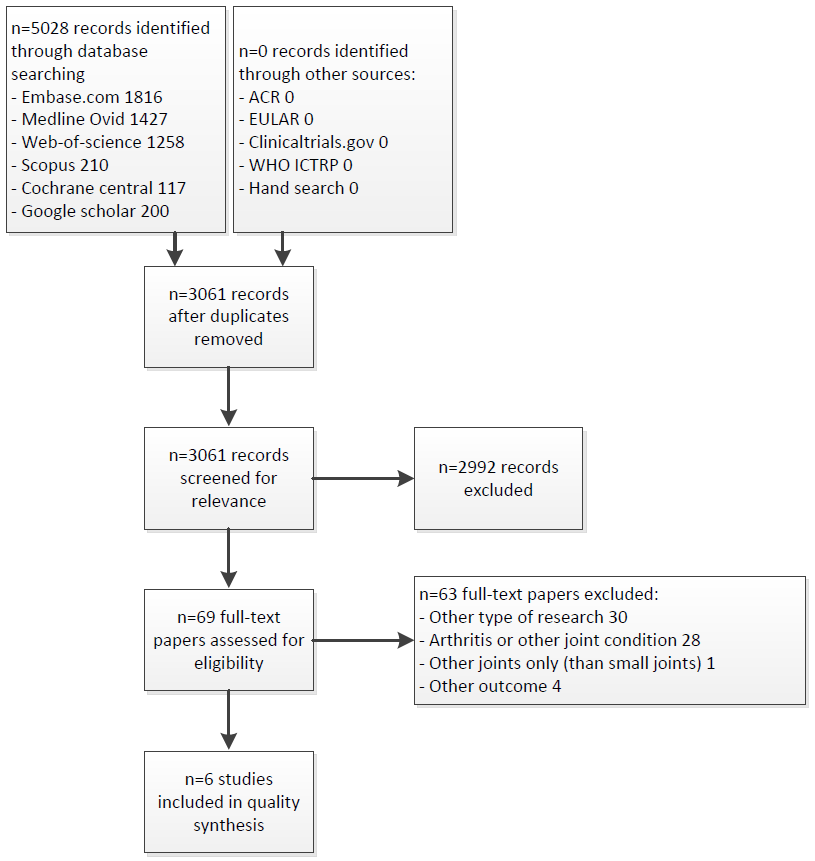


**Table S1: Best evidence synthesis used in this review**

| **Evidence level** | **Criteria** |
| --- | --- |
| Strong evidence | Provided by statistically significant findings in multiple high-quality studies |
| Moderate evidence | Provided by statistically significant findings in 1 high-quality study and in multiple low-quality studies |
| Limited evidence | Provided by statistically significant findings in 1 or more low-quality studies |
| Indicative findings | Provided by statistically significant findings, irrespective of study quality |
| No or insufficient evidence | In the case that results of eligible studies do not meet the criteria for one of the above stated levels of evidence or in the case of conflicting (statistically significant positive and statistically significant negative) results or in the case of no eligible studies |

**Table S2: Criteria used for the assessment of quality of included studies**

| **Item** | **Criteria** |
| --- | --- |
| 1 | Definition of study population  Sufficient description of characteristics of population  *A ‘1’ is given if a paper describes at least setting and time period of the study, ages of patients (and its range) and man:woman ratio* |
| 2 | Definition of outcome  Definition of arthritis is given |
| 3 | Selection bias  Clear description of selection of study subjects.  *A ‘1’ is given if a paper describes how study subjects were selected (description of in- and exclusion criteria) from the population level to the study level* |
| 4  5  6 | Follow-up  Organization of follow-up  *A ‘1’ was given if a structured follow-up was applied (not only on patients request)*  Follow up time ≥ 1 years  *One year was set as an arbitrary acceptable duration of follow-up to measure the outcome*  Lost to follow-up  *(quantitative and qualitative) information about withdrawals was presented* |
| 7 | Assessment of outcome  Presence of arthritis is assessed via clinical examination by a rheumatologist |
| 8  9  10  11 | Analysis and data presentation  Frequencies of most important prognostic factors were given  Frequencies of outcomes were given  Unadjusted (univariable) estimates were presented  Adjusted (multivariable) estimates were presented |
| **12**  **13**  **14**  15  16  17  18 | US acquisition  Standardized joint examination  *According to international recognized recommendations*  Definition of included pathology  *According to international recognized scoring systems*  Use of (at least) middle or (better) high-class US machine with high-resolution linear transducers  Inclusion of the two US modes  *B-mode (or greyscale) and Doppler mode (color or power Doppler)*  Inclusion of tenosynovitis  Reliability data on US performance  *Inter-, intra-reader agreement (ICC/Kappa statistics ≥0.6)*  Inclusion of findings in healthy subjects and correcting for it in the analyses |

Criteria 12-14 are mandatory (indicated in blue).

A study was considered to be of high-quality if the total quality score was >= 80.6% (median of the quality scores obtained in this review) and if all 3 mandatory criteria were fulfilled.

**Table S3: Results of the quality assessment scores of the included studies**

|  | **Criteria** | | | | | | | | | | | | | | | | | |  |
| --- | --- | --- | --- | --- | --- | --- | --- | --- | --- | --- | --- | --- | --- | --- | --- | --- | --- | --- | --- |
| **Author, year [ref]** | **1** | **2** | **3** | **4** | **5** | **6** | **7** | **8** | **9** | **10** | **11** | **12** | **13** | **14** | **15** | **16** | **17** | **18** | **Total score** |
| van de Stadt, 2010 [11] | 1 | *1* | 1 | *1* | 0 | 0 | *1* | 1 | 1 | 1 | 0 | *1* | 1 | 1 | 1 | 1 | 1 | 0 | 14/18=77.8 |
| Pratt, 2013 [8] | 1 | 1 | *0* | 0 | 1 | 0 | 1 | 1 | 1 | 0 | 1 | 1 | 1 | 1 | 1 | 0 | 1 | 0 | 12/18=66.7 |
| Rakieh, 2015 [10] | 1 | 1 | 1 | 1 | 1 | 1 | 1 | 1 | 1 | 1 | 1 | *1* | 1 | 1 | 0 | 0 | 1 | 0 | 15/18=83.3^ǂ^ |
| Nam, 2016 [9] | 1 | 1 | 1 | 1 | 1 | *1* | 1 | 1 | 1 | 1 | 0 | 1 | 1 | 1 | 1 | 0 | *1* | 0 | 15/18=83.3^ǂ^ |
| Zufferey, 2017 [6] | 1 | 1 | *1* | 0 | 1 | 0 | 1 | 1 | 1 | 0 | 1 | *1* | 1 | 1 | 0 | 1 | 1 | 1 | 11/18=61.1 |
| van der Ven, 2017 [7] | 1 | 1 | 1 | 1 | 1 | 1 | 1 | 1 | 1 | 1 | 1 | 1 | 1 | 1 | 1 | 0 | 0 | 0 | 15/18=83.3^ǂ^ |

1=present; 0=absent or no information. Scores solved by discussion represented in Italics. ^ǂ^ High-quality study.

**Table S4: Calculated Positive Predictive value (PPV), Negative Predictive Value (NPV), and increase in absolute risks**

| **Author, year** | **Percentage**  **Arthritis development(%)** | **PPV (%) (95% CI)** | **Increase in probability of arthritis development (PPV minus pretest probability) (%)*** | **Absence of arthritis developemnt (1 minus prevalence)(%)** | **NPV (%) (95% CI)** | **Increase in probability of absence of arthritis development (NPV minus (1 minus prevalence)) (%)*** |
| --- | --- | --- | --- | --- | --- | --- |
| Rakieh et al. 2015 [10] | 50.0 | 66.7 (52.1-78.6) | 16.7 | 50.0 | 58.2 (51.1-65.0) | 8.2 |
| Nam et al. 2016 [9] | 41.9 | GSUS≥2: 47.8 (42.2-53.5)  PDUS≥1: 75.0 (53.6-88.6) | GSUS≥2: 5.9  PDUS≥1: 33.1 | 57.1 | GSUS≥2: 70.5 (57.9-80.5)  PDUS≥1: 63.8 (59.9-67.5) | GSUS≥2: 12.4  PDUS≥1: 5.7 |
| van der Ven et al. 2017 [7] | 17.8 | Positive US: 27.4 (20.2-36.1)  PDUS≥1: 34.6 (20.7-51.8) | Positive US: 9.6  PDUS≥1: 16.8 | 82.2 | Positive US: 87.5 (82.4-91.3)  PDUS≥1: 85.1 (81.9-87.9) | Positive US: 5.3  PDUS≥1: 2.9 |
| van de Stadt et al. 2010 [11] | 23.4 | GSUS synovitis: 29.2 (15.4-48.2)  PDUS: 30.3 (18.3-45.8)  GSUS effusion: 36.4 (20.4-56.0)  Tenosynovitis: 30.8 (12.6-57.9) | GSUS synovitis: 5.8  PDUS: 6.9  GSUS effusion: 13.0  Tenosynovitis: 7.4 | 76.6 | GSUS synovitis: 77.4 (74.9-79.7)  PDUS: 78.0 (74.9-80.8)  GSUS effusion: 78.2 (75.7-80.6)  Tenosynovitis: 77.1 (75.3-78.8) | GSUS synovitis: 0.8  PDUS: 13.9  GSUS effusion: 1.6  Tenosynovitis: 0.5 |
| Pratt et al. 2013 [8] | 42.7 | a: 68.4 (61.5-74.6)  b: 70.5 (62.8-77.3)  c: 71.9 (64.3-78.5)  d: 69.1 (61.2-76.0)  e: 75.0 (63.9-83.6) | a: 25.7  b: 27.8  c: 29.2  d: 26.4  e: 32.3 | 57.3 | a: 71.1 (67.2-74.8)  b: 68.9 (65.4-72.2)  c: 69.8 (66.22-73.2)  d: 68.0 (64.6-71.3)  e: 63.8 (61.3-66.2) | a: 13.8  b: 11.6  c: 12.5  d: 10.7  e: 6.5 |
| Zufferey et al. 2017 [6] | 8.8 | SONAR >8/22: 23.5 (12.1-40.9)  US score ≥2 joints with grade ≥2 synovitis: 25.0 (14.8-39.0) | SONAR >8/22: 14.7  US score ≥2 joints with grade ≥2 synovitis: 16.2 | 91.2 | SONAR >8/22: 95.2 (89.4-97.9)  US score ≥2 joints with grade ≥2 synovitis: 96.7 (89.9-99.0) | SONAR >8/22: 4.0  US score ≥2 joints with grade ≥2 synovitis: 5.5 |

*Caution with interpretation due to lack of confidence intervals.

Studies marked in grey are scored as high-quality study (high-quality study >80% (which is the median of all quality scores)).

GSUS=greyscale ultrasound; NPV=negative predictive value; PDUS=Power Doppler ultrasound; PPV=positive predictive value; US=ultrasound

a. GSUS sum score ≥2; b. GSUS sum score/6 joints (worst hand) ≥2; c. GSUS number of joints ≥1: ≥3. d. PDUS sum score ≥1; e. PDUS number of joints ≥1: ≥2

**File S1: Search strategies for each database**

**Embase.com**

('rheumatoid arthritis'/exp OR (((rheumat* OR deforman* OR inflammat*) NEAR/3 arthrit*) OR (chronic* NEAR/3 (polyarthrit* OR poly-arthritis)) OR rheumarthrit* OR (ra AND (rheum* OR arthrit* OR joint*)) OR (Juvenile NEAR/3 Idiopath* NEAR/3 Arthrit*)):ab,ti) AND (echography/exp OR echograph/exp OR ultrasound/exp OR (echogra* OR ultraso* OR (US NEAR/3 (examin* OR finding* OR result* OR imag* OR assess* OR scor* OR parameter*)) OR sonogra*):ab,ti) AND ('differential diagnosis'/exp OR 'validation process'/exp OR 'validation study'/exp OR 'predictive value'/exp OR 'diagnostic error'/exp OR reliability/exp OR reproducibility/exp OR 'scoring system'/exp OR 'observer variation'/exp OR 'diagnostic value'/exp OR validity/exp OR 'instrument validation'/exp OR 'diagnostic test accuracy study'/exp OR 'sensitivity and specificity'/exp OR (((different* OR improve* OR essential* OR tool* OR value* OR impact* OR perform* OR earl* OR pitfall* OR error*) NEAR/6 (diagnos* OR detect* OR identif* OR predictiv*)) OR (false NEXT/1 (positive* OR negative*)) OR undiagnos* OR undetect* OR valid* OR score OR scores OR scoring OR accura* OR reliab* OR utilit* OR ((observer* OR intraobserver* OR interobserver* OR reader* OR intrareader* OR interreader*) NEAR/3 (varia* OR bias)) OR sensitivit* OR specificit* OR reproducib*):ab,ti) NOT ([Conference Abstract]/lim OR [Letter]/lim OR [Note]/lim OR [Editorial]/lim) AND [english]/lim

**Medline Ovid**

(exp "Arthritis, Rheumatoid"/ OR (((rheumat* OR deforman* OR inflammat*) ADJ3 arthrit*) OR (chronic* ADJ3 (polyarthrit* OR poly-arthritis)) OR rheumarthrit* OR (ra AND (rheum* OR arthrit* OR joint*)) OR (Juvenile ADJ3 Idiopath* ADJ3 Arthrit*)).ab,ti.) AND (exp Ultrasonography/ OR Ultrasonography.xs. OR Ultrasonics/ OR (echogra* OR ultraso* OR (US ADJ3 (examin* OR finding* OR result* OR imag* OR assess* OR scor* OR parameter*)) OR sonogra*).ab,ti.) AND ("Diagnosis, Differential"/ OR "Validation Studies"/ OR exp "Diagnostic Errors"/ OR "Reproducibility of Results"/ OR exp "sensitivity and specificity"/ OR (((different* OR improve* OR essential* OR tool* OR value* OR impact* OR perform* OR earl* OR pitfall* OR error*) ADJ6 (diagnos* OR detect* OR identif* OR predictiv*)) OR (false ADJ (positive* OR negative*)) OR undiagnos* OR undetect* OR valid* OR score OR scores OR scoring OR accura* OR reliab* OR utilit* OR ((observer* OR intraobserver* OR interobserver* OR reader* OR intrareader* OR interreader*) ADJ3 (varia* OR bias)) OR sensitivit* OR specificit* OR reproducib*).ab,ti.) NOT (letter OR news OR comment OR editorial OR congresses OR abstracts).pt. AND english.la.

**Cochrane central**

((((rheumat* OR deforman* OR inflammat*) NEAR/3 arthrit*) OR (chronic* NEAR/3 (polyarthrit* OR poly-arthritis)) OR rheumarthrit* OR (ra AND (rheum* OR arthrit* OR joint*)) OR (Juvenile NEAR/3 Idiopath* NEAR/3 Arthrit*)):ab,ti) AND ((echogra* OR ultraso* OR (US NEAR/3 (examin* OR finding* OR result* OR imag* OR assess* OR scor* OR parameter*)) OR sonogra*):ab,ti) AND ( (((different* OR improve* OR essential* OR tool* OR value* OR impact* OR perform* OR earl* OR pitfall*) NEAR/6 (diagnos* OR detect* OR identif* OR predictiv*)) OR undiagnos* OR undetect* OR valid* OR score OR scores OR scoring OR accura* OR reliab* OR utilit* OR ((observer* OR intraobserver* OR interobserver* OR reader* OR intrareader* OR interreader*) NEAR/3 (varia* OR bias)) OR sensitivit* OR specificit*):ab,ti)

**Web-of-science**

TS=(((((rheumat* OR deforman* OR inflammat*) NEAR/3 arthrit*) OR (chronic* NEAR/3 (polyarthrit* OR poly-arthritis)) OR rheumarthrit* OR (ra AND (rheum* OR arthrit* OR joint*)) OR (Juvenile NEAR/3 Idiopath* NEAR/3 Arthrit*))) AND ((echogra* OR ultraso* OR (US NEAR/3 (examin* OR finding* OR result* OR imag* OR assess* OR scor* OR parameter*)) OR sonogra*)) AND ( (((different* OR improve* OR essential* OR tool* OR value* OR impact* OR perform* OR earl* OR pitfall*) NEAR/6 (diagnos* OR detect* OR identif* OR predictiv*)) OR undiagnos* OR undetect* OR valid* OR score OR scores OR scoring OR accura* OR reliab* OR utilit* OR ((observer* OR intraobserver* OR interobserver* OR reader* OR intrareader* OR interreader*) NEAR/3 (varia* OR bias)) OR sensitivit* OR specificit*))) AND LA=(english) AND DT=(article)

**Scopus**

TITLE-ABS-KEY(((((rheumat* OR deforman* OR inflammat*) W/3 arthrit*) OR (chronic* W/3 (polyarthrit* OR poly-arthritis)) OR rheumarthrit* OR (ra AND (rheum* OR arthrit* OR joint*)) OR (Juvenile W/3 Idiopath* W/3 Arthrit*))) AND ((echogra* OR ultraso* OR (US W/3 (examin* OR finding* OR result* OR imag* OR assess* OR scor* OR parameter*)) OR sonogra*)) AND ( (((different* OR improve* OR essential* OR tool* OR value* OR impact* OR perform* OR earl* OR pitfall*) W/6 (diagnos* OR detect* OR identif* OR predictiv*)) OR undiagnos* OR undetect* OR valid* OR score OR scores OR scoring OR accura* OR reliab* OR utilit* OR ((observer* OR intraobserver* OR interobserver* OR reader* OR intrareader* OR interreader*) W/3 (varia* OR bias)) OR sensitivit* OR specificit*))) AND Language(english) AND doctype(ar)

**Google scholar**

"rheumatoid arthritis"|rheumarthritis echography|echogram|ultrasonography|ultrasonogram|ultrasound "differential|early diagnosis"|"diagnostic|predictive tool|value|impact|performance|pitfall"|validity|accuracy|reliability|utility |sensitivity|specificity
